# Predicting College Students’ Bike-Sharing Intentions Based on the Theory of Planned Behavior

**DOI:** 10.3389/fpsyg.2022.836983

**Published:** 2022-03-03

**Authors:** Xiaofang Chen

**Affiliations:** School of Arts, Zhejiang University of Finance and Economics, Hangzhou, China

**Keywords:** bike-sharing, theory of planned behavior, perceived benefits, government policy, sustainable bike-sharing

## Abstract

Shared bicycles are sustainable and effective transportation tools in college campuses. Accordingly, this study aimed to assess the behavioral intention of college students toward bike-sharing as an environmentally friendly and social mode of travel. It applied the Theory of Planned Behavior framework to a bike-sharing context and explored the impact of perceived benefits and government policy on college students’ bike-sharing usage. A survey of 934 college students was conducted in Zhejiang province to test the proposed model, and 782 were valid. The findings pointed out that attitude, subjective norms, and perceived behavioral control have a significant and positive impact on college students’ intentions toward bike-sharing. Meanwhile, the empirical results revealed that perceived benefits and government policy were the important factors driving college students’ intention and behavior for bike-sharing usage. Moreover, the results ascertained that the intention was aligned with actual actions; eventually, some targeted managerial implications are presented. This study enhances the current understanding of the usage behavior of college students in bike-sharing and provides timely insights for government policymakers and enterprise operators to promote sustainable bike-sharing practices in China and other countries.

## Introduction

As a promising new travel mode, bike-sharing has become increasingly popular all over the world in places, such as Australia, Netherlands, Singapore, Italy, and the United Kingdom. In China, bike-sharing has become the third largest mode of public transportation (Gu et al., 2019). It has been widely recognized that the value of bike-sharing is beneficial with regard to psychological, educational, social, relaxation, physiological, and esthetic aspects (Zhou et al., 2020). Bike-sharing has also become a popular travel option for many people including college students in China. According to the Ministry of Transport’s statistics, the number of shared bicycles in China has reached 19.45 million, and the average daily order exceeded 45.7 million in 2020 (Ministry of Transport, China, 2020).

Along with the development and intensification of Chinese public services and facilities, various green travel alternatives are emerging, like car-sharing and bike-sharing. Although the use of electric vehicles (EVs) is increasing, it is insufficient to deal with the challenges of sustainability. On the one hand, EVs can alleviate the amount of greenhouse gas emissions but cannot mitigate traffic congestion. On the other hand, affording the cost of energy cars is a big issue for college students. So, because of the characteristics of low-carbon and environmental protection, bike-sharing has been considered as the fourth largest means of transportation after cars, busses, and subways in China. It also improves travel efficiency and provides a green and economic travel choice for college students.

However, bike-sharing also has its disadvantages; in particular, bikes cannot carry loads and bike riding is not friendly to rainy days. Other factors, including possible malfunctions, significant physical effort and lower speed also limit the distance that college students can travel. As for college students, there is no school-exclusive software app or campus bike-sharing service platform. Meanwhile, the mechanical failures and aging of accessories reduce the bicyclists’ satisfaction.

In general, social psychologists believe that most people’s behaviors are goal-oriented, and the implementation of such behaviors will occur with the pre-design (Ajzen, 1991). When factors that affect intentions are identified, people’s intentions and even actual behaviors can be changed (Holdsworth et al., 2019). Therefore, it is necessary to identify the factors that contribute more to the intention of bike-sharing as people’s transportation modes. According to the Theory of Planned Behavior (TPB), a widely recognized theoretical model for behavior analysis, people’s intentions can be predicted using structural equation modeling (SEM) and multiple-group analysis (Ajzen, 1991; Wang et al., 2018). So far, a few studies have measured users’ behavioral intention toward bike-sharing by adopting the TPB (Kaplan et al., 2015; Han et al., 2017; Cai et al., 2018). These prior works have investigated bike-sharing behavior and its influencing factors from the perspective of individual behavior; specifically, these factors were mainly focused on user wellbeing (Ma et al., 2018) or group differences (Buck et al., 2013). However, empirical evidence regarding the factors driving sustainable usage behavior of shared bicycles from the perspective of perceived benefits and government policy is lacking.

In fact, effective government policies will promote the use of bicycles (Gatersleben and Appleton, 2007). Meanwhile, to alleviate the disadvantages of bike-sharing, more attention needs to be paid to the perceived benefits of bike-sharing. Thus, in order to solve urban traffic problems and increase user satisfaction, the current study adopts and extends the TPB framework by enhancing the variables of perceived benefits and governmental policy to investigate the bike-sharing usage of college students in Zhejiang province, China. This study aims to comprehensively understand college students’ special willingness and motivation toward bike-sharing and reveal the relationship among various factors in the extended TPB, so as to form a low-carbon and environment-friendly travel mode among college students, and boost the social communication of college students. Specifically, whether the perceived benefits and the governmental policy have influenced the bike-sharing usage and what shaped college students’ bike-sharing intentions are subsequently addressed.

The remainder of this paper begins with a literature review in the first section and hypothesis development is presented in the second section. In the third section, a description of the research methodology and an analysis of the data are presented, followed by a description of the results and an analysis of the discussion. Finally, this study concludes by summarizing the findings, implications, and the limitations and future work.

## Literature Review and Research Hypotheses

### Reviews on Theory of Planned Behavior Related to Bike-Sharing

The Theory of Planned Behavior (TPB) is a theory of social psychology that focuses on the determinants of an individual’s behavior (Ajzen, 1991), which can be traced back to the Theory of Multi-Attribute Attitude (Fishbein, 1963). It is believed that behavioral intention is the direct factor that determines behavior and is affected by behavior attitude and subjective norms. The TPB involves three factors, i.e., the attitude, subjective norms, and perceived behavioral control. According to Ajzen (2011), the more positive the attitude, the greater the support of related persons and the stronger the control of perceptual behavior, the greater intention to perform a given behavior, and vice versa. Over the past decades, the TPB has been widely used in various fields including traffic (Cai et al., 2018), environmental protection (Groot and Steg, 2010), and cycling (Donald et al., 2014). Some studies have also utilized TPB to explain bike-sharing intentions and behaviors (Kaplan et al., 2015).

Notably, attitude refers to an individual’s judgment of a particular behavior (Ajzen, 1991) and also refers to their feelings or emotions toward a certain behavior (Sun et al., 2019), such as cycling (Donald et al., 2014). The more favorable attitudes that are shown toward a certain behavior, the greater the likelihood that an individual will commit it (Willis et al., 2015). Some researchers have indicated that there existed a positive relationship between the pleasurable attitudes and the cycling (Kaplan et al., 2015). Therefore, attitude plays an important role in the selection of bike-sharing as an activity or transport mode (Sun et al., 2019). The attitude is positively beneficial to the adoption of bike-sharing intentions (Chen and Lu, 2016a; Wang et al., 2018).

Perceived behavioral control (PBC) is an important factor that refers to the degree of ease or difficulty perceived by a person when performing a certain behavior (Ajzen, 1991). It is also recognized that PBC is responsible for the valuable information regarding the actual strength and is considered as “an additional direct predictor of behavior” (Ajzen, 2002), which means people’s behavior is strongly influenced by their confidence in their ability to perform it. For example, some researchers found that a high level of PBC leads to a stronger behavioral intention toward bike-sharing (Kaplan et al., 2015), that is, PBC is sufficient to account for bike-sharing intentions.

Subjective norms represent a kind of pressure from other social groups that are important to them (e.g., friends, family, and peers of an individual; Ajzen, 1991). For example, if bike-sharing is misunderstood as a “stupid act” by a group of college students, it may prevent other college students from preferring bike-sharing behavior. Subsequently, some studies have demonstrated that subjective norms impact civilized cycling intentions and actual behaviors (Acheampong, 2017).

Behavioral intention refers to the personal tendency and motivation of an individual to do something, reflecting one’s willingness to pay the time, expense, and effort to accomplish something (Ajzen, 1991; Cai et al., 2018). As a central factor, intentions are assumed to influence a behavior. In order to perform the behavior, people will make their effort on what they are planning to exert. The intention to engage in a behavior will make its performance realized. In other words, the stronger the willingness of a certain intention, the more possible it will be achieved. Thus, the following hypotheses are developed as:

*H1:* Attitude significantly and positively influences college students’ bike-sharing intention.

*H2:* Perceived behavioral control significantly and positively influences college students’ bike-sharing intention.

*H3:* Subjective norms significantly and positively influence college students’ bike-sharing intention.

*H4:* The bike-sharing intention significantly and positively influences college students’ bike-sharing behavior.

### Reviews on Perceived Benefits Research Related to Bike-Sharing

According to Ajzen (1991), additional variables can be added to the TPB when they are empirically found to be significant to the original constructs. For example, the perceived benefits (Willis et al., 2015), which are generally associated with perceived value and behavioral intentions (Pourabedin et al., 2015), can be added, because the perception of the benefits of bike-sharing not only affects the intention to cycle for transportation but also influences a college student’s decision on selecting bike-sharing. Perceived benefits have been widely recognized as an important factor in behavioral analysis. For example, perceived usefulness and perceived ease of use were added in the technology acceptance model (TAM) to examine users’ willingness of bike-sharing (Chen and Lu, 2016a).

Bike-sharing can promote college students’ health (Fuller et al., 2013); one example of this is reducing obesity (Frank et al., 2010). The calorie consumption of bike-sharing riding is relatively high. Based on the TAM, the perceived health of bike-sharing positively affects ones’ attitudes (Ma et al., 2019). To some extent, the health benefits of bike-sharing would outweigh the risks of collisions and exposure to air pollution (De Hartog et al., 2010). Bike-sharing can also bring economic benefits (Sahlqvist and Heesch, 2012). The lower the travel cost, the greater is the willingness to choose bike-sharing (Akar and Clifton, 2009). Bike-sharing can influence the social relationship (Fraboni et al., 2016). In our previous work (Chen et al., 2020), the authors explored the college students’ social relationship in usage of social software, which could be enhanced through usage of bike-sharing in this study. Bike-sharing can also enable college students to effectively avoid traffic congestion (Bopp et al., 2012). Since it has been launched around colleges, bike-sharing riding has become one of the most convenient forms of transportation for college students because of the flexibility of departure time (Akar and Clifton, 2009). Bike-sharing can provide people with a convenient travel mode (Poveda-Reyes et al., 2021), which not only helps college students form positive attitudes toward bike-sharing, but also facilitates them to travel whenever and wherever needed. Hence, the following hypotheses are developed as:

*H5:* Perceived benefits significantly and positively influence college students’ bike-sharing attitude.

*H6:* Perceived benefits significantly and positively influences college students’ perceived behavioral control on bike-sharing.

*H7:* Perceived benefits significantly and positively influence college students’ subjective norms on bike-sharing.

*H8:* Perceived benefits significantly and positively influence college students’ bike-sharing intention.

### Reviews on Governmental Policy Research Related to Bike-Sharing

According to the theory of acceptance and use of technology (UTAUT) that contains the TPB, social influence and facilitating conditions are important variables to influence ones’ intentions and behaviors (Gao et al., 2019). In this study, government policy is defined as policies and regulations that will be successful in promoting users’ transformation of intention to behaviors (Sun et al., 2019). As we all know, government policies can make many changes to regional traffic. In order to construct a good traffic environment, the government often implements a series of measures, such as introducing pedestrian areas and safe residential streets for cyclists. Bike-sharing is a shared model that enables citizens to obtain a shared fleet of bicycles from a mobile app or a membership in an urban area. It can change people’s attitude and influence each other, and finally facilitate people to work, travel, and go to school, so bike-sharing is considered as a successful “last mile” solution.

The government’s incentive effect on busses or the control of private cars plays an important role in the choice of the travel mode. For example, in the United States, green traffic regulations have been implemented, such as the improvement and promotion of green travel rates, especially the public transport travel rate (Cervero et al., 2004). In China, in order to encourage bicycle riding, the government reduced the bicycle use restrictions and cycling barriers; subsequently, bike-sharing has been carried forward as one of the government’s initiative policies to encourage cycling behaviors (Chen and Lu, 2016a). On 1 May 2008, the Hangzhou municipal government in China launched the first information technology-based public bike-sharing program.

With the influence of the government policy, it was found that bicyclists care about the environment more than non-bicyclists (Emond and Handy, 2012), because bike-sharing can reduce traffic congestion (Cai et al., 2018) and alleviate noise and air pollution (Groot and Steg, 2010). When PBC is realistic, it can be used to predict the probability of a successful behavioral action. So, when policymakers invest more resources in strengthening the bike-sharing infrastructure, for instance, creating a bicycle-sharing lane network, adopting traffic calming measures on various roads, and providing priority for certain intersections for college students, it may influence college students’ attitude toward bike-sharing and generate beliefs, intentions, and behaviors on bike-sharing. Hence, the following hypotheses are developed as:

*H9:* Governmental policy significantly and positively influences college students’ bike-sharing attitude.

*H10:* Governmental policy significantly and positively influences college students’ perceived behavioral control on bike-sharing.

*H11:* Governmental policy significantly and positively influences college students’ subjective norms on bike-sharing.

*H12:* Governmental policy significantly and positively influences college students’ bike-sharing intention.

Consequently, considering that the impact of perceived benefits and government policies on bike-sharing intention has not been comprehensively addressed in the literature, meanwhile, as a special group, how to improve the perceived benefits of bike-sharing to meet the demand of college students has not been addressed. What measures government can take has not been addressed either. It is worthwhile to provide insights into their potential influence on attitude, subjective norms, perceived behavioral control, and bike-sharing intention. Therefore, the perceived benefits and government policies are added to the TPB in this study as two new variables to comprehensively investigate college students’ bike-sharing intentions.

## Methodology and Data

### The Scheme of the Research Design

Structural equation modeling (Marcoulides, 1998; Wang et al., 2018) is one of the most salient research methods across a variety of disciplines, such as e-commerce (Chong, 2013), marketing (Hair et al., 2012), and tourism (Zhou et al., 2020). Partial least squares-structural equation modeling (PLS-SEM) is a composite-based approach to SEM that uses linear combinations of indicator variables in the structural model (Rigdon et al., 2017). Other statistical methods such as t-test should consider whether the theory is contradicted, and whether the corresponding model is consistent with the theory, and vice versa. In contrast, these difficulties do not arise in PLS-SEM, because it only requires constructing structural connections between variables. Therefore, PLS-SEM offers more flexibility when it comes to formative measures (Hair et al., 2012).

**Figure 1:**
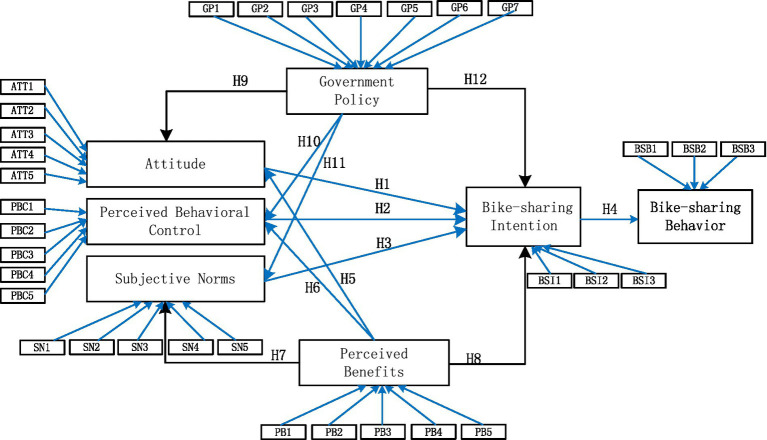
Integrated theoretical framework.

Figure 1 depicts the proposed integrated modeling framework by incorporating all the hypotheses that have been developed. PLS-SEM is adopted in this study, because it can explain the variance of the endogenous constructs and cope with highly complex models (Hair et al., 2012) through the following two-step approach: (1) the evaluation of the measurement models and (2) the assessment of the structural model.

To test the theoretical model, the following procedures were implemented. First, the discriminant validity and composite reliability were confirmed by implementing the confirmatory factor analysis (CFA). Second, by using SPSS v20, the demographics and relevant frequency statistics analysis was carried out to cluster college students into different groups. Next, the PLS-SEM was employed to investigate and verify the hypotheses for the factors and the relationships among latent constructs. Finally, the data analysis was conducted by Smart PLS 3 software on the proposed model.

### Data Collection

The data source was made up of three parts. First, the evolutionary bike-sharing process was constructed with the relevant literature. Second, a rational questionnaire was designed through structured interviews. The interviewers were mainly college students from undergraduate to master students in Zhejiang province, China. The number of the interviewers was 32. Subsequently, each interview lasted more than 30 min and was either face to face or *via* telephone. The whole set of questions or concerns is shown in Appendix (See Appendix). Third, the questionnaire was developed by adapting from relevant literature review, and finalizing the interview feedback and proposed hypothesis.

To ensure that the questionnaire was acceptable and reasonable, a review panel of five professors held symposiums and advised modifications to the items. Then, the final version of the online survey was handed out to more than 1,300 college students who were randomly selected. Notably, online surveys would help to avoid college students from submitting incomplete or repeated surveys.

The questionnaire was implemented *via* the utilization of “Questionnaire Star” (also known as Sojump), which is the largest online survey portal in China, and involves more than 127.1 million questionnaires. It has collected more than 10.099 billion answers since its launch in 2006.^1^ Among the 1,300 visits, a total of 934 college students responded to the questionnaire, with a response rate of 72.5%. Among the 934 responses, because some of the questionnaires answered were either too short (e.g., only finished within less than 30 s) or not complete, 152 were obsolete, and 782 were valid, which corresponds to an effective response rate of 83.73%. The survey includes two parts, with the first one involving the demographic information of respondents, and the second part involving the relationships among five independent variables and two dependent variables in the proposed model.

### The Social Economic Characteristics of the Sample

As shown in Table 1, of all the respondents, the ratio of two groups (i.e., male and female) of college students was comparatively balanced, accounting for 45.8 and 54.2%, respectively. Most of them were undergraduates; in particular, a small proportion was master students. Most of the respondents were familiar with bike-sharing, while only a few of whom did not know it. It was found that most of the respondents considered bike-sharing useful, only few of them were uncertain of its usefulness. Regarding the usage frequency of bike-sharing by different groups of college students, it was found the various groups used bike-sharing differently. Given the statistics mentioned in Table 1, it is apparent that bike-sharing has been considered as an indispensable part of public transportation by college students.

**Table 1:** Demographics and relevant frequency statistics.

**Measure**	**Item**	**Count**	**Percentage (%)**
Gender	Male	358	45.8
	Female	424	54.2
Grades	Freshman	389	49.7
	Sophomore	142	18.2
	Junior	98	12.5
	Senior	35	4.5
	Master	99	12.7
	Others	19	2.4
Familiarity with bike-sharing	Quite a lot	173	22.1
	Generally	593	75.8
	None	16	2
Evaluation of bike-sharing	Useful	658	84.1
	A little useful	108	13.8
	Useless	7	0.9
	Uncertain	9	1.2
Frequency of bike-sharing	Daily	248	31.7
	Weekly	357	45.7
	Monthly	130	16.6
	Never	47	6

### Assessment of the Measurement Model

Referring to Table 2, five independent variables (i.e., attitude, subjective norms, perceived behavioral control, perceived benefits, and government policy) and two dependent variables (i.e., bike-sharing intention, and behavior) were elaborated in the proposed model. By taking “attitude” as an illustrative example, which is one of the TPB’s factor, the five items (ATT1–ATT5) can be obtained according to two references (i.e., Kaplan et al., 2015; Acheampong, 2017) and also verified through the interviews. The reliability and validity were enhanced to measure the CFA by adding multiple choice questions or measurement items relevance from different sides to each variable (Chong, 2013). To ensure that college students would answer the questions honestly and seriously, similar questions were answered by the respondents. Then, the responses that answered the similar questions contradictorily were filtered.

**Table 2:** Survey items related to behavioral intention toward the sustainable bike-sharing usage.

**Variable**	**Items**	**References**
**Attitude (ATT)**		Kaplan et al. (2015) and Acheampong (2017)
ATT1	I think using bike-sharing is a good sport	
ATT2	I like to use bike-sharing	
ATT3	I think it’s a good idea to use a bike-sharing	
ATT4	I have a good feeling about using bike-sharing as a means of traveling	
ATT5	I feel a sense of caring toward bike-sharing	
**Subjective Norms (SN)**		Ajzen (1991) and Sun et al. (2019)
SN1	Most people who are important to me think I should use bike-sharing	
SN2	Most people who are familiar to me think I should use bike-sharing	
SN3	Most people who are important to me would want me to use bike-sharing	
SN4	People whose opinions I value would prefer that I should use bike-sharing	
SN5	My friends’ positive opinion influences me to use bike-sharing	
**Perceived Behavioral Control (PBC)**		Han et al. (2017) and Si et al. (2020)
PBC1	I think it is good to use bike-sharing	
PBC2	I think that I am confident to use bike-sharing	
PBC3	I think that I have the ability to use bike-sharing	
PBC4	I think that using the bike-sharing is totally within my control	
PBC5	I can participate in the decision-making process of using bike-sharing	
**Perceived Benefits (PB)**		Pourabedin et al. (2015) and Willis et al. (2015)
PB1	I think it is economical to use bike-sharing	
PB2	I think it is convenient to use bike-sharing	
PB3	I think the use of bike-sharing can reduce traffic congestion	
PB4	I think the use of bike-sharing benefits the physical exercise	
PB5	I think the use of bike-sharing can save time	
**Government Policy (GP)**		Gatersleben and Appleton (2007) and Sun et al. (2019)
GP1	The government’s promotional policy made me willing to use bike-sharing	
GP2	The government’s political support made me willing to use bike-sharing	
GP3	The government’s call made me willing to use bike-sharing	
GP4	The separated bikeway made me willing to use bike-sharing	
GP5	The secure parking made me willing to use bike-sharing	
GP6	The good road surface quality made me willing to use bike-sharing	
GP7	The calmness of the traffic made me willing to use bike-sharing	
**Bike-Sharing Intention (BSI)**		Wang et al. (2018) and Si et al. (2020)
BSI1	I intend to use bike-sharing as my own willingness	
BSI2	I intend to use bike-sharing in campus	
BSI3	I intend to use bike-sharing more than other travel tools	
**Bike-Sharing Behavior (BSB)**		Bamberg (2012) and Cai et al. (2018)
BSB1	I have used bike-sharing whenever I need it	
BSB2	I have used bike-sharing wherever I need it	

The variables were measured with a five-point Likert type scale, ranking from strongly disagree (i.e., 1) to strongly agree (i.e., 5). The descriptive statistics on structural equations are available in Table 3.

**Table 3:** Descriptive statistics and the output of the measurement model.

**Indicators**	**FL**	**CR**	**AVE**	***α***	**Indicators**	**FL**	**CR**	**AVE**	***α***
**ATT**		0.869	0.572	0.947	**SN**		0.975	0.885	0.979
ATT1	0.744				SN1	0.945			
ATT2	0.842				SN2	0.961			
ATT3	0.744				SN3	0.958			
ATT4	0.720				SN4	0.954			
ATT5	0.724				SN5	0.884			
**PBC**		0.870	0.573	0.957	**PB**		0.855	0.541	0.936
PBC1	0.703				PB1	0.735			
PBC2	0.712				PB2	0.710			
PBC3	0.803				PB3	0.782			
PBC4	0.773				PB4	0.704			
PBC5	0.787				PB5	0.745			
**GP**		0.936	0.675	0.945	**BSI**		0.871	0.692	0.949
GP1	0.826				BSI1	0.838			
GP2	0.832				BSI2	0.800			
GP3	0.862				BSI3	0.857			
GP4	0.835				**BSB**		0.788	0.561	0.839
GP5	0.822				BSB1	0.845			
GP6	0.798				BSB2	0.806			
GP7	0.773				BSB3	0.802			

*FL, Factor loadings; CR, Composite reliability; AVE, Average variance extracted; *α*, Cronbach’s alpha; ATT, Attitude; PBC, Perceived behavioral control; SN, Subjective norms; GP, Government policy; PB, Perceived benefits; BSI, Bike-sharing intention; and BSB, Bike-sharing behavior*.

A total of 33 measurement questions were categorized to measure the variables in this study. The construction validity can be achieved by establishing convergent and discriminant validity (Hair et al., 2010). Reliability related to the internal consistency of multiple metrics can be used to measure each variable (Fornell and Larcker, 1981). The assessment of reflective measurement models involves evaluating the reliability of the measures (i.e., indicator reliability and internal consistency reliability) and the validity (i.e., convergent and discriminant validity).

## Results

### Results From the Measurement Model

PLS-SEM contains the outer model evaluation and the inner model evaluation (Hair et al., 2012). Outer model assessment involves three aspects, i.e., individual indicator reliabilities, the reliabilities for each construct’s composite of measures, and the measures’ convergent and discriminant validities (Hair et al., 2012). Assessment of reflective outer models involves the internal consistency reliability (composite reliability), convergent validity [average variance extracted (AVE)], and discriminant validity (Fornell-Larcker criterion; cross-loadings). If the evidence of reliability and validity can be testified by the outer model, it is then appropriate to examine the inner model estimates, such as the coefficient of determination (*R*^2^).

Referring to Table 3, the reliability and validity of the collected data were confirmed by the CFA, followed by the modeling and hypothesis testing. It is found that all factor loadings of the indicators are higher than 0.7, proving the validity of the constructs according to Si et al. (2020). All the composite reliability (CR) values are higher than 0.7, proving the reliability of internal consistency according to Fornell and Larcker (1981). The AVE is a reasonable method to reflect the convergence between groups of items in the potential structure. It is found all AVE values are larger than 0.5, proving the convergent validity according to Chen and Lu (2016a). The Cronbach’s alpha values of attitude, subjective norms, perceived behavioral control, perceived benefits, government policy, bike-sharing intention, and behavior are 0.947, 0.979, 0.957, 0.936, 0.945, 0.949, and 0.839 respectively, which are all higher than 0.7, indicating the reliability of all indicators according to Hair et al. (2010), thus specifying the accuracy of the measures and the alleviation of random errors.

Additionally, all square roots of variables’ AVEs are higher than all the correlation coefficients between variables, and the discriminant validity of this model data is obvious according to Fornell and Larcker (1981). The results of the discriminant validity measures are shown in Table 4.

**Table 4:** Result of discriminant validity measures.

**Variable**	**ATT**	**BSB**	**BSI**	**GP**	**PB**	**PBC**	**SN**
ATT	**0.792**						
BSB	0.781	**0.749**					
BSI	0.747	0.603	**0.832**				
GP	0.662	0.638	0.565	**0.822**			
PB	0.713	0.626	0.795	0.633	**0.736**		
PBC	0.782	0.644	0.827	0.607	0.760	**0.757**	
SN	0.582	0.594	0.585	0.535	0.562	0.548	**0.941**

*ATT, Attitude; PBC, Perceived behavioral control; SN, Subjective norms; GP, Government policy; PB, Perceived benefits; BSI, Bike-sharing intention; and BSB, Bike-sharing behavior. Diagonal elements are the square root of AVE with the bolded number*.

The correlation analysis focuses on the degree of changes among the variables. It is found that the correlation coefficients among all variables are between 0.535 and 0.827, indicating a high and positive correlation among variables, and proving the validity of the preliminary hypotheses. The results of the testing of hypotheses are presented in Table 5.

**Table 5:** Result of the hypotheses testing.

**Hypotheses**	**Path**	**Mean (M)**	**T statistics**	**STDEV**	**Remarks**	**Result**
**TPB**
H1	ATT → BSI	0.145	3.086	0.045	^***^	Accept
H2	PBC → BSI	0.466	6.487	0.073	^***^	Accept
H3	SN → BSI	0.137	5.248	0.026	^***^	Accept
H4	BSI → BSB	0.903	40.861	0.008	^***^	Accept
**Perceived benefits influences**						
H5	PB → ATT	0.657	18.908	0.035	^***^	Accept
H6	PB → PBC	0.795	32.435	0.025	^***^	Accept
H7	PB → SN	0.372	9.203	0.040	^***^	Accept
H8	PB → BSI	0.729	24.096	0.030	^***^	Accept
**Government policy influences**						
H9	GP → ATT	0.246	6.445	0.038	^***^	Accept
H10	GP → PBC	0.103	4.145	0.025	^***^	Accept
H11	GP → SN	0.301	6.831	0.044	^***^	Accept
H12	GP → BSI	0.104	3.511	0.029	^***^	Accept

*STDEV, Standard deviation; ATT, Attitude; PBC, Perceived behavioral control; SN, Subjective norms; GP, Government policy; PB, Perceived benefits; BSI, Bike-sharing intention; and BSB, Bike-sharing behavior*.

*** *T-statistics > 2.580 of significance at 1% and passes the *t*-test at a 99% confidence interval*.

### Results From the Structural Model

**Figure 2:**
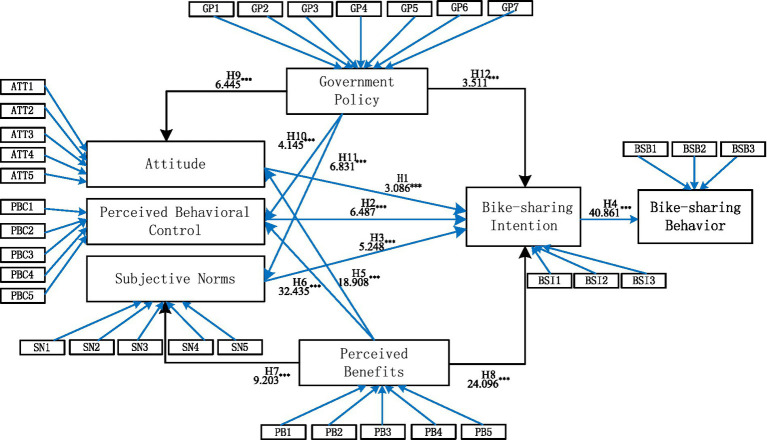
The result of the structural model tests. (*** means T-statistics > 2.580 of significance at 1% and passes the *t*-test at a 99% confidence interval).

Generally, PLS-SEM can be used to verify the fitness of the research model and the corresponding hypotheses. Based on the CFA analysis elaborated above, fitness among various factor dimensions was examined, showing the internal consistency reliability and the discriminant validity of the structural model. It is also important to test the fitness of five independent variables and two dependent variables, and the results of the structural model tests are shown in Figure 2.

Referring to Table 5, in terms of the influence of attitude on the bike-sharing intention, the value of *t* is 3.086 > 2.580, which indicates that attitude influences bike-sharing intention positively and significantly, verifying H1. The results also indicate that the value of *t* of perceived behavioral control on bike-sharing intention is 6.487 > 2.580, thus verifying H2. This confirms that the perceived behavioral control influences the bike-sharing intentions of college students significantly. Then, the value of *t* of subjective norms on the bike-sharing intention is 5.248 > 2.580, thus supporting H3. This result implies that social pressure also influences the bike-sharing intentions of college students. Furthermore, the value of *t* of the bike-sharing intention on bike-sharing behavior is 40.861 > 2.580, and H4 is accepted consequently. It is also found that the perceived benefits and government policy significantly and positively influence the bike-sharing intention. Consequently, H5 through H12 was verified.

Furthermore, it is worth mentioning that the t-values of perceived benefits for H5, H6, H7, and H8 are very high, compared to those of government policy, indicating that perceived benefits have a very meaningful impact on the interpretation of college students’ bike-sharing intentions.

## Discussion

From the perspective of the extended TPB model, the perceived benefits and government policy are explored as two new variables to investigate bike-sharing among college students in Zhejiang province, China. It also provides a reference for the formulation of long-term bike-sharing policies in other regions. The findings pointed out three main theoretical implications: (1) attitude, perceived behavioral control, and subjective norms positively influence bike-sharing intention and behavior; (2) perceived benefits can enhance college students’ preferences for bike-sharing; and (3) government policy significantly influences the use of bike-sharing by college students. Referring to Table 6, the empirical results are found as follows:

1. Attitude, perceived behavioral control, and subjective norms positively influence bike-sharing intention;
2. Bike-sharing intention positively and significantly affects the practical bike-sharing behavior;
3. Perceived benefits, which is an important factor in the extended TPB model, can enhance college students’ preferences for bike-sharing; and
4. Government policy significantly affects the use of bike-sharing by college students.

**Table 6:** Summary of the empirical results.

**Hypotheses**	**Path**	**Effect?**		**Significant?**	
		**Positive**	**Negative**	**Yes**	**No**
**Finding 1**					
H1	ATT → BSI	√		√	
H2	PBC → BSI	√		√	
H3	SN → BSI	√		√	
H4	BSI → BSB	√		√	
**Finding 2**					
H5	PB → ATT	√		√	
H6	PB → PBC	√		√	
H7	PB → SN	√		√	
H8	PB → BSI	√		√	
**Finding 3**					
H9	GP → ATT	√		√	
H10	GP → PBC	√		√	
H11	GP → SN	√		√	
H12	GP → BSI	√		√	

*ATT, Attitude; PBC, Perceived behavioral control; SN, Subjective norms; GP, Government policy; PB, Perceived benefits; BSI, Bike-sharing intention; and BSB, Bike-sharing behavior*.

The results of this study are consistent with the previous TPB findings. Specifically, attitudes toward the advantages gained by cycling are identified to directly impact bicycling behavior. As one kind of bicycle transportation, bike-sharing is affected by personal attitudes, which have a close relationship with bicycle use, including preferences for cycling activity (Handy et al., 2010). It is also found that travelers whose purpose is to go to school are more willing to choose bike-sharing than those whose purpose is to go to work or play (Liu et al., 2021).

However, it is claimed that attitude alone is not sufficient to predict individual intentions (Floress et al., 2017), so the perceived benefits variable, based on its instrumental function, is added to the TPB in this study to further investigate the bike-sharing selection by college students. In contrast, bike-sharing has some disadvantages, for example, availability of too many brands of shared bicycles, poor quality, and destruction possibility, which cannot be solved by college students themselves, so effective policies and administrative supervision on bike-sharing need to be adopted by the government. For example, the government can urge bike-sharing enterprises to improve people’s satisfaction with bike-sharing’ usage, such as improving the devices and equipment of shared bicycles. The government can also provide some incentives to encourage bike-sharing usage to increase the utilization rate of shared bicycles. The empirical results show that the perceived benefits and government policy have a positive effect on college students’ attitudes toward bike-sharing.

The empirical results also show that perceived benefits and government policy significantly and positively influence perceived behavioral control. It was found that an individual’s perception of cycling was of great significance in deciding how to select transportation tools (Ajzen, 1991). For example, people may worry about traffic jams, lack of safety protection, unpredictable weather, and likelihood of bicycles being stolen when they use shared bicycles, as do college students. Meanwhile, psychological theories have pointed out that perceived behavioral control is a key factor in changing people’s intention to engage in healthy behaviors, such as cycling (Chen and Lu, 2016a). When bike-sharing is advocated by the government policy, it will promote the preference of college students for bike-sharing because of lowered usage barriers.

The empirical results also show that perceived benefits and government policy significantly and positively influence subjective norms. Studies have suggested that people’s subjective norms toward bicycling are strong (Kaplan et al., 2015), because the subjective norms of bicyclists have a significant influence on their loyalty to shared bicycles (Chen and Lu, 2016b). Therefore, due to the encouragement of government policies on bike-sharing, college students will influence each other and exhibit a preference for shared bicycles. Moreover, the perceived benefits of bike-sharing mean that more convenience is brought to college students, and it is beneficial to use shared bicycles.

Bike-sharing intentions affect bike-sharing behavior significantly. In China, bike-sharing services has spread rapidly; also, the increased use of bicycles has been attributed to commuting among college districts (Whannell et al., 2012). Due to the perceived benefits, an increasing number of college students are satisfied with bike-sharing. This is conducive to forming an environmentally friendly travel mode for college students. Therefore, government should strengthen the management power on a high level of attributes to increase college students’ perceived usefulness to the environment and their environmentally friendly intentions. Moreover, the government should attach importance to the implementation of bike-sharing, thus encouraging and increasing bicycle ridership (Larsen and El-Geneidy, 2011). The government can also contribute to reducing the cycling barriers. In general, targeted policies can be adopted to encourage college students’ preferences for bike-sharing. Presently, bike-sharing has also played a new role in transforming individual cycling behaviors into socialized behaviors (Liu et al., 2021), which facilitates new forms of travel behavior among college students. For example, the “Singles Day,” the festival advocated by Ofo, the top bike-sharing business in China, has shown the social attributes of bike-sharing, which satisfies cyclists’ social interests to a certain extent. In many brands of shared bicycles, social networking, competition, and entertainment have been integrated into a comprehensive service platform to establish a social network for young people.

## Conclusion and Future Work

### Conclusion

In the big data era, the various forms of the sharing economy are driven by the “Internet +” environment. Bike-sharing, as one new type of sharing economy, is also influenced by “Internet.” Therefore, the “Internet + bicycle” with low-carbon travel model has become an effective form of the country’s innovative development, which needs the support of college students as well (Liu et al., 2021). In this study, key findings and the implications are listed in Table 7 and are detailed as follows:

1. It is suggested that enterprises develop a school-exclusive software app and establish a campus bike-sharing service platform, where bike-sharing is limited to teachers and students on campus. Meanwhile, enterprises can enhance their campus cards with the function of bike-sharing usage to waive the deposits of bike-sharing. Apparently, improving the satisfaction of bike-sharing is important, and it is demanded to promote the perceived benefits of bike-sharing for college students, which is conducive to developing their intention and behavior of bike-sharing.
2. It is suggested that the government should call on college students to pay attention to environmental concerns, which may trigger attitudes, willingness, and behaviors toward sustainable transportation usage. It was found that protecting the environment from people’s own point of view was important, so promoting the connection between values, attitudes, behavior, and the use of green travel—such as bike-sharing—is meaningful. Bike-sharing can reduce overall air pollution levels (De Hartog et al., 2010), and reduce noise pollution and waste gas (Cai et al., 2018). To some extent, the perceived green value has improved the pleasure of using bike-sharing, so bike-sharing is conducive to forming a low-carbon and environment-friendly travel mode.
3. It has been found that bike-sharing has become a new form of social behavior of college students, whose social interaction demands can be met through a bike-sharing platform. Establishing a bike-sharing club can not only provide a platform to promote college students’ health during cycling but also create an atmosphere for mutual exchange. Cycling reflects a mixture of self-interest and prosocial motives, which promote college students to choose bike-sharing as both social and environmental behavior. Meanwhile, compared with other sports, bike-sharing is considered as a rational, sustainable, and effective activity among school districts. Hence, bike-sharing is conducive to boosting the social communication of college students.
4. It is also found that the government policy can be formulated to influence college students’ choices of a travel mode. For example, when the government policy increases the barriers to private car or promotes social riding facilities, it will change travelers’ choice of a travel mode. Additionally, laws and regulations can be formulated to regulate bike-sharing behaviors, such as individual integrity points, which can be exchanged for the riding time. Because the government policy has a positive influence on environmental decision making (Sun et al., 2019), the support of government policy is conducive to encouraging college students’ green mode of transport.

**Table 7:** Summary of the key findings and practical implications.

**S. No.**	**Results/Key findings**
1.	Promoting the perceived benefits of bike-sharing for college students is conducive to developing their intention and behavior of bike-sharing
2.	Bike-sharing is conducive to forming a low-carbon and environment-friendly travel mode
3.	Bike-sharing is conducive to boosting the social communication of college students
4.	The support of government policy is conducive to encouraging college students’ green mode of transport
**Practical implications**
1.	To develop the school-exclusive software App
2.	To call on college students to use bike-sharing
3.	To establish a bike-sharing club as a hobby on campus
4.	To make policies to assist the development of the bike-sharing

### Future Work

Despite the existence of certain valuable research, some limitations remained in this study.

First, while attitude, perceived behavior control, subjective norms, perceived benefits, and government policy have been explored in the extended TPB model, other social factors like habits, social environment, and psychological factors are ignored. Subsequently, this modeling issue may also have potential impacts on college students’ bike-sharing intention. Meanwhile, this study mainly focused on the personal benefits, but it is meaningful to study and compare the influence of both personal benefit and community benefit in the future work. Given this, future studies need to include other important factors in the extended TPB model (or the UTAUT model) to more comprehensively examine the bike-sharing intention of college students.

Second, in order to capture the impacts of TPB more comprehensively and dynamically on bike-sharing intentions and behaviors, there needs to be a longitudinal analysis over different periods (Chen and Lu, 2016a). However, the data collected in this research restrict such an analysis; therefore, future studies need to examine the bike-sharing intentions and behaviors through a longitudinal analysis and minimize the unexplained variance in the data set. Meanwhile, the data in this study were collected from college students in certain colleges, which may lead to biased results and hinder the generalizability of the findings. So, future studies need to further examine and validate our findings at different universities in different countries during different periods.

Finally, although the TPB and its extended model have been used to examine college students’ bike-sharing intentions, the PLS-SEM is mainly implemented with the static process analysis and fail to explore the dynamic decision-making process. Therefore, in future work, more in-depth studies or behavioral experimental methods can be explored to examine the influence of various variables on college students’ bike-sharing intentions and behaviors.

## Data Availability Statement

The raw data supporting the conclusions of this article will be made available by the authors, without undue reservation.

## Ethics Statement

Ethical review and approval was not required for the study on human participants in accordance with the local legislation and institutional requirements. Written informed consent for participation was not required for this study in accordance with the national legislation and the institutional requirements.

## Author Contributions

The author confirms being the sole contributor of this work and has approved it for publication.

## Funding

This work was supported by the Humanities and Social Sciences Research Project of Education Ministry of China (no. 19JDSZ3003).

## Conflict of Interest

The author declares that the research was conducted in the absence of any commercial or financial relationships that could be construed as a potential conflict of interest.

## Publisher’s Note

All claims expressed in this article are solely those of the authors and do not necessarily represent those of their affiliated organizations, or those of the publisher, the editors and the reviewers. Any product that may be evaluated in this article, or claim that may be made by its manufacturer, is not guaranteed or endorsed by the publisher.

## Footnotes

^1^ https://www.wjx.cn/html/aboutus.aspx

## Appendix

### Interviews with 32 college students using bike-sharing are as follows:

1. How do you know about bike-sharing?

2. How often do you use bike-sharing (daily, weekly, monthly, or never)?

3. How long do you feel about using bike-sharing (all the time or not)?

4. What are the advantages of bike-sharing?

5. What is your attitude toward bike-sharing?

6. What is your purpose of using bike-sharing?

7. What factors do you think will affect your use of bike-sharing?

8. What is your attitude towards the price of bike-sharing (expensive or cheap)?

9. How do you think about the environmental protection of bike-sharing (environmentally friendly or not)?

10. How do you think about the safety of bike-sharing (safe or dangerous)?
